# Model-Driven Discovery of Highly Sensitive Nanocolumnar NiO Electrochemical Glucose Sensors

**DOI:** 10.1021/acsengineeringau.6c00026

**Published:** 2026-07-16

**Authors:** MohammadAli Maleki Bigdeli, Jennifer Bruce, Abebaw B. Jemere, Kenneth D. Harris, Wylie Stroberg

**Affiliations:** † Department of Mechanical Engineering, 3158 University of Alberta, Donadeo Innovation Centre for Engineering, 116 St NW, Edmonton, Alberta T5K 1S6, Canada; ‡ Quantum and Nanotechnologies Research Centre, 103212 National Research Council Canada, 11421 Saskatchewan Dr NW, Edmonton, Alberta T6G 2M9, Canada; § Department of Chemical and Materials Engineering, 3158 University of Alberta, Donadeo Innovation Centre for Engineering, 116 St NW, Edmonton, Alberta T5K 1S6, Canada; ∥ Department of Biomedical Engineering, 3158 University of Alberta, Donadeo Innovation Centre for Engineering, 116 St NW, Edmonton, Alberta T5K 1S6, Canada

**Keywords:** Nonenzymatic sensing, Reaction−diffusion modeling, Glucose sensing, Electrochemical sensors, Glancing angle deposition (GLAD), Kinetic Monte Carlo (kMC) simulation

## Abstract

Nanostructured NiO electrodes fabricated using glancing angle deposition (GLAD) are promising platforms for nonenzymatic electrochemical glucose sensing, owing to their high surface area and tunable morphology. With GLAD, adjusting the deposition angle and substrate rotation rate can significantly affect electrochemical performance; however, identifying optimal GLAD structures for specific use cases is still largely experimental, relying on trial and error. Here, we develop a multiscale modeling framework that links the GLAD film growth process to glucose electro-oxidation performance by combining on-lattice kinetic Monte Carlo (kMC) simulations with morphology characterization and a coupled reaction–diffusion electrochemical model. Key morphological features, including surface area, porosity, and directional tortuosities, are quantified from kMC-generated structures across a wide range of GLAD geometries, such as slanted posts, helical shapes, and vertical nanocolumns. These features are then incorporated into a homogenized porous electrode model for glucose electro-oxidation on Ni-based catalysts. The model clarifies how the trade-off between surface area and mass transport governs electrode sensitivity. We identify a slanted-post morphology deposited at 72.5° as the optimal design for maximizing glucose electro-oxidation, and we find that physical electrodes formed with similar geometries were approximately 25% more sensitive to glucose than those formed with the vertical post morphology previously considered to be the optimum (i.e., 1.38 mA/mM·cm^2^ vs 1.12 mA/mM·cm^2^). Beyond glucose, this modeling workflow provides general guidelines for designing GLAD-fabricated electrodes for other biosensing targets and for electrochemical applications, including energy conversion and hydrogen generation.

## Graphical abstract

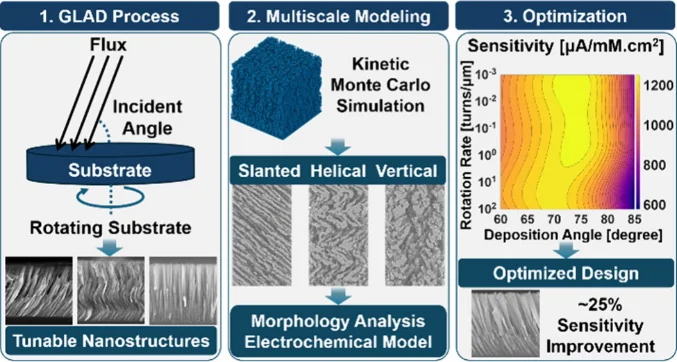

## 1 Introduction

Continuous and accurate monitoring of glucose concentration in the human body is crucial for the effective management of diabetes and other related disorders.^1,2^ Traditional glucose monitoring typically involves finger-prick capillary blood sampling.^3^ This method does not monitor blood sugar level continuously; therefore, to follow blood sugar level over time, repeated finger-prick tests are required, which can be uncomfortable, particularly for children and elderly individuals. Moreover, this method is significantly affected by user error, which can reduce measurement accuracy.^3,4^ To overcome these limitations, semi-invasive and noninvasive electrochemical glucose biosensors have emerged as alternative approaches for measuring glucose concentrations.^5^ Semi-invasive methods, such as percutaneous and subcutaneous electrochemical glucose biosensors, require device implantation and are often associated with challenges related to long-term stability, biocompatibility, and patient comfort.^6^ On the other hand, noninvasive wearable glucose biosensors have gained attention as they can monitor glucose and other diabetes-related biomolecules through biofluids like sweat, eliminating the need for implantation, minimizing patient discomfort, and enabling continuous long-term monitoring.^7–9^ However, a significant limitation is the relatively low concentration of glucose in biofluids like sweat as compared to blood, which necessitates highly sensitive detection strategies.^10^

Electrochemical biosensors, which have the potential for high sensitivity, can be categorized as enzymatic or nonenzymatic.^11^ Enzymatic biosensors use specific enzymes as receptors, making them highly selective, but also prone to the environment’s local thermal and chemical conditions. They are often costly and may degrade over time or when stored under nonideal conditions.^11,12^ Therefore, considerable research has focused on the development of nonenzymatic biosensors, in which nanostructured electrodes mimic the catalytic function of enzymes (also known as “nanozymes”).^12,13^ Material composition plays an important role in the performance of these nonenzymatic biosensors. Among various material candidates for glucose detection,^14,15^ Ni-based biosensors have demonstrated strong performance due to Ni’s exceptional catalytic activity for oxidation reactions in alkaline media.^16^ Moreover, Ni is highly abundant, implying that cost may not be excessive.

A biosensor’s structural design can strongly influence overall performance via factors such as porosity, active surface area, and mass transport properties.^17–19^ Using nanozymes, highly porous electrodes with high surface area can be employed to improve detection. Consequently, nonenzymatic biosensors offer a promising approach for developing wearable, noninvasive glucose monitors for analysis of sweat samples.^20,21^ Various methods^22–26^ have been developed to fabricate nanozyme-based electrodes, and glancing angle deposition (GLAD) has recently emerged as a promising, high-performance technique (Figure 1).^27–32^

**Figure 1:**
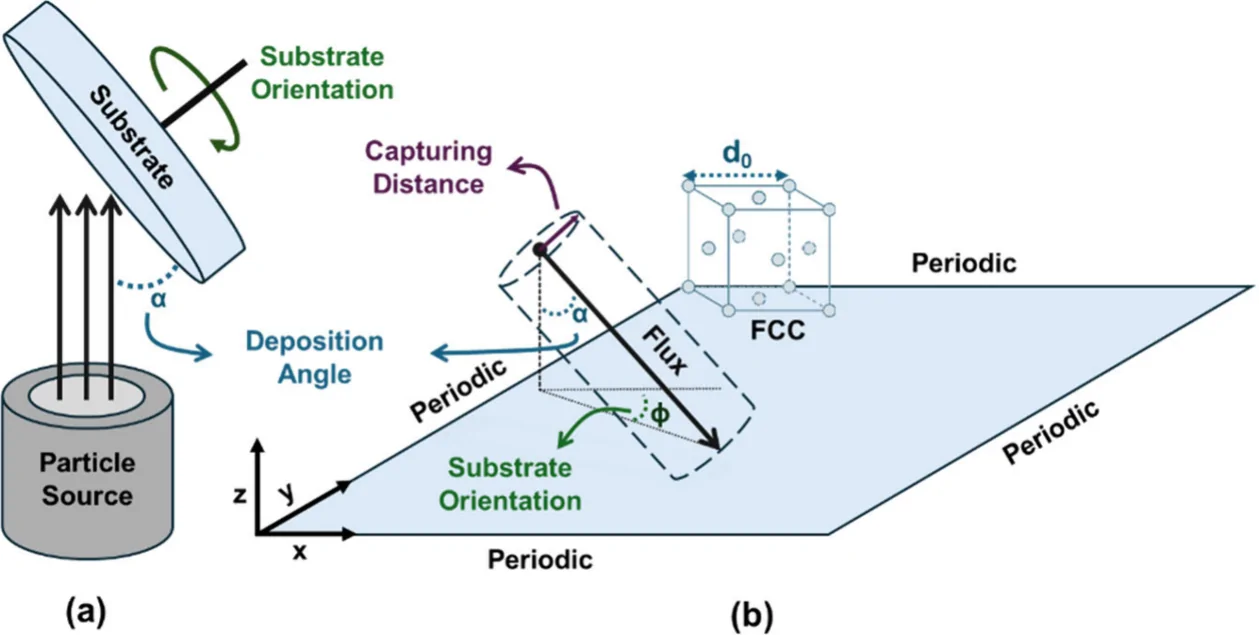
a) Schematic diagram of the glancing angle deposition technique, and b) schematic representation of the deposition process in the on-lattice GLAD kMC model. A random point at the top of the domain is selected as the initial position, and the particle follows the indicated trajectory according to eq (15) until it is captured by the growing film. Periodic boundary conditions are applied on the sides of the domain in the x and y directions.

GLAD is a physical vapor deposition technique that enables precise control over thin film nanostructure morphology by dynamically adjusting the deposition angle and substrate rotation rate during deposition. In this technique, the target material is deposited onto a substrate positioned at an oblique angle, and the resulting interatomic shadowing conditions lead to the formation of highly porous columnar structures.^32^ By incorporating substrate rotation during the GLAD process, a wide range of nanostructures, including slanted and vertical posts, helical structures, and more complex morphologies, can be fabricated.^33–40^ Moreover, adjusting the deposition angle allows for precise control over morphological features such as porosity, surface area, column orientation, and film thickness.^32^ This morphological tailorability makes GLAD-fabricated structures promising for electrochemical applications, particularly in sensing devices. Recently, highly porous GLAD-fabricated electrodes with large surface areas have been applied to nonenzymatic glucose detection in sweat and serum.^16,31^ These electrodes have demonstrated excellent performance, exhibiting high sensitivity, selectivity, and reproducibility, and it was clearly shown that variations in GLAD parameters modulate device performance.^16,31,41^ However, despite these investigations, experimentally identifying the optimal GLAD-fabricated structures for enhanced electrode performance remains challenging as it is a labor and time-intensive process.

For glucose sensing, several studies have reported GLAD-fabricated electrodes using different materials and morphologies;^16,31,42–44^ however, these studies have used a range of different deposition angles and substrate rotation rates, resulting in different designs, and no guideline currently exists that systematically connects these parameters to electrochemical performance. Therefore, identifying the optimal GLAD-fabricated structures for enhanced electrode performance remains challenging as it is a labor and time-intensive process, requiring several days to perform all the preparation, fabrication and testing steps to analyze a single parameter set. Consequently, modeling approaches offer a valuable alternative, enabling much more rapid analysis of how variations in deposition angle and substrate rotation rate influence both the morphological characteristics and the overall electrochemical performance of the electrodes.

Our previous numerical studies of 2D^19^ and 3D GLAD structures^17^ demonstrated that mass-transport limitations significantly affect electrode performance. Our analysis revealed how porosity and surface area vary with deposition angle for both slanted and vertical posts, and we found that, although the maximum surface area of GLAD structures occurs at deposition angles of 60–65°, structures fabricated at a 72.5° deposition angle exhibited the highest glucose electro-oxidation rates due to more efficient mass transport. While these studies provided valuable insights into the impact of mass transport limitations on glucose electro-oxidation in GLAD structures, the analysis was based entirely on geometrical considerations. In that work, an explicit electrochemical model was not developed, the influence of incremental changes in the substrate rotation rate on sensor performance was not determined, and, very critically, no experimental validation was performed.

In the present study, we fill these gaps by developing an integrated electrochemical model of glucose electro-oxidation on Ni-based GLAD-fabricated electrodes by combining a dual-path kinetic model^45^ with mass-transport and charge-transfer effects (Figure 2). Using a surrogate model trained on a data set of 270 kMC simulated GLAD structures (spanning deposition angles from 50° to 85° in 2.5° increments and various substrate rotation rates), we quantify key GLAD geometric parameters, including porosity, surface area, and directional tortuosities. The geometric features are then incorporated into a homogenized electrochemical model to analyze how GLAD morphology governs glucose electro-oxidation and to identify the optimal electrode geometry. To validate the predictive power of the model, we directly compare modeling results with experimental data from GLAD electrodes fabricated at different deposition angles and substrate rotation rates (from no rotation to high-speed rotation), providing robust support for the simulation outcomes and creating physical devices with glucose sensitivity ∼ 25% greater than the previously established peak values. Importantly, this work clarifies how mass transport affects the performance of GLAD-fabricated electrochemical sensors. Although we focus on glucose electro-oxidation, the same modeling framework can be applied to other analytes (e.g., lactate) and to energy applications such as hydrogen generation in GLAD-based systems. It also provides a modeling approach that reduces reliance on trial-and-error experimental optimization.

**Figure 2:**
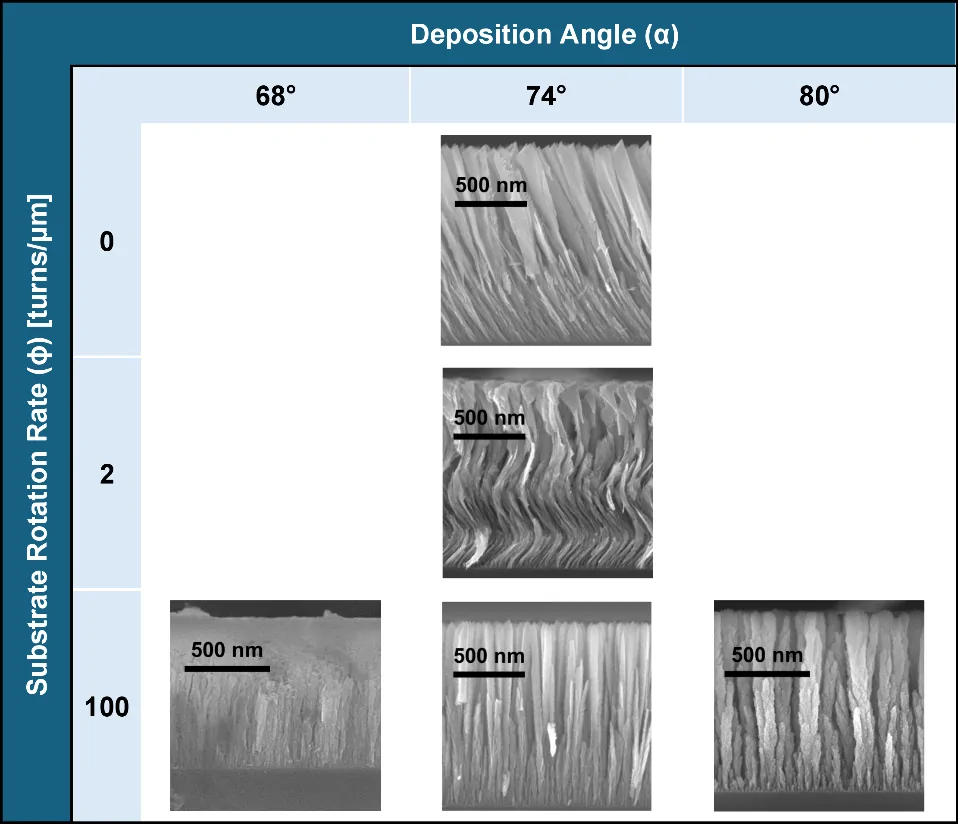
Cross-sectional SEM images of GLAD-fabricated electrodes at different deposition angles (α = 68°, 74°, and 80°) and substrate rotation rates (ϕ = 0, 2, and 100 turns/μm).

## 2 Methodology

### 2.1 Dual-Path Glucose Electro-Oxidation Kinetics

In our electrochemical model, we employ a dual-path mechanism reported previously^45^ for glucose electro-oxidation on Ni­(OH)_2_/NiOOH catalysts in an alkaline electrolyte. The purpose of this section is to briefly describe the adopted mechanism and the associated rate expressions used in the governing equations.

Glucose oxidation is coupled to the Ni^2+^/Ni^3+^ redox transition. Prior to the sensing experiment, GLAD NiO structures form Ni­(OH)_2_ in an electrochemical activation process in alkaline conditions. Under alkaline conditions and at elevated potential, Ni­(OH)_2_ is oxidized to NiOOH, which is the catalytic agent responsible for the nonenzymatic glucose oxidation, as shown in the following reaction,

1 $$\mathrm{Ni}{\left(\mathrm{OH}\right)}_{2}+{\mathrm{OH}}^{-}\overset{k_{f}}{\underset{k_{b}}{⇌}}\mathrm{NiOOH}+H_{2}O+e^{-}$$

Glucose oxidation follows a dual-path mechanism. In the first pathway, glucose oxidation proceeds via an Eley–Rideal (ER)^46^ path in which glucose reacts directly with NiOOH to form an adsorbed intermediate, which is subsequently oxidized in the presence of additional NiOOH to yield the product:

2 $$\mathrm{NiOOH}+\mathrm{glucose}\overset{k_{g}}{\to }\mathrm{Ni}{\left(\mathrm{OH}\right)}_{2}\text{‐}{\left(\mathrm{intermediate}\right)}_{\mathrm{ads}}$$

3 $$\mathrm{Ni}{\left(\mathrm{OH}\right)}_{2}\text{‐}{\left(\mathrm{intermediate}\right)}_{\mathrm{ads}}+\mathrm{NiOOH}\overset{k_{p}}{\to }\mathrm{product}+2\mathrm{Ni}{\left(\mathrm{OH}\right)}_{2}$$

Because glucose does not need to adsorb prior to reaction, this pathway remains active even when adsorption sites are limited and, therefore, contributes significantly at higher anodic potentials.

In the second pathway, a Langmuir–Hinshelwood (LH) type pathway, glucose first adsorbs onto available Ni­(OH)_2_ surface sites. The adsorbed glucose then reacts with neighboring NiOOH to form an adsorbed intermediate, which is subsequently oxidized to the final product:

4 $$\mathrm{Ni}{\left(\mathrm{OH}\right)}_{2}+\mathrm{glucose}\overset{k_{a}}{\underset{k_{d}}{⇌}}\mathrm{Ni}{\left(\mathrm{OH}\right)}_{2}-{\left(\mathrm{glucose}\right)}_{\mathrm{ads}}$$

5 $$\mathrm{Ni}{\left(\mathrm{OH}\right)}_{2}\text{‐}{\left(\mathrm{glucose}\right)}_{\mathrm{ads}}+\mathrm{NiOOH}\overset{k_{\mathrm{int}}}{\to }\mathrm{Ni}{\left(\mathrm{OH}\right)}_{2}\text{‐}{\left(\mathrm{intermediate}\right)}_{\mathrm{ads}}+\mathrm{Ni}{\left(\mathrm{OH}\right)}_{2}$$

6 $$\mathrm{Ni}{\left(\mathrm{OH}\right)}_{2}\text{‐}{\left(\mathrm{intermediate}\right)}_{\mathrm{ads}}+\mathrm{NiOOH}\overset{k_{p}}{\to 2}\mathrm{Ni}{\left(\mathrm{OH}\right)}_{2}+\mathrm{product}$$

Because this pathway requires both adsorbed glucose and NiOOH to be present on the surface, it is most active near the Ni^2+^/Ni^3+^ redox potential.

#### 2.1.1 Rate Expression

To translate the dual-path mechanism into an electrochemical model, we define rate expressions for the Ni redox step and for the reactions in both pathways. The surface coverages of Ni­(OH)_2_, NiOOH, adsorbed glucose and adsorbed intermediate are denoted by θ_Ni(OH)_2__, θ_NiOOH_, θ_g_, and θ_int_, respectively. Site conservation is enforced through,^45^

7 $$\theta_{\mathrm{Ni}{\left(\mathrm{OH}\right)}_{2}}+\theta_{\mathrm{NiOOH}}+2\theta_{g}+2\theta_{\mathrm{int}}=1$$

where the factors of 2 reflect that glucose and the intermediate each occupy two sites.

The Ni^2+^/Ni^3+^ redox kinetics are modeled using a Butler–Volmer expression^47^ that accounts for both forward oxidation of Ni­(OH)_2_ and backward reduction of NiOOH. It is driven by the local overpotential η = *E*_app_ – ψ – E_eq_, where *E*_app_ is the applied electrode potential, ψ is the local electrolyte potential and E_eq_ is the equilibrium potential of the Ni^2+^/Ni^3+^ redox pair. The redox rate (r_e_) is then given by

8 $$r_{e}=k_{f}\exp\left(\frac{\beta F\eta }{\mathrm{RT}}\right)\theta_{\mathrm{Ni}{\left(\mathrm{OH}\right)}_{2}}-k_{b}\exp\left(-\frac{\left(1-\beta \right)F\eta }{\mathrm{RT}}\right)\theta_{\mathrm{NiOOH}}$$

Here *k*_f_ and *k*_b_ are the forward and backward rate constants for the Ni redox step, β is the charge transfer coefficient, F is the Faraday constant, R is the gas constant, and T is the absolute temperature.

The glucose reaction rates associated with the dual-path mechanism are defined in Table 1. Here, r_g_ and r_p_ represent the solution-phase oxidation route (ER pathway), while r_a_, r_int_, r_p_ and r_d_ describe adsorption, intermediate oxidation, surface reaction, and desorption steps associated with the LH pathway. *k*_a_, *k*_d_, *k*_g_, *k*_int_ and *k*_p_ are the corresponding kinetic rate constants, and C_g_ is the glucose concentration in the solution.

**Table 1:** Rate Expressions Describing Dual-Path (Eley-Rideal/Langmuir-Hinshelwood) Glucose Electro-Oxidation on Ni­(OH)_2_/NiOOH Catalysts

**Expression**	**Description**	Path
r_a_ = *k*_a_C_g_θ_Ni(OH)_2__^2^	Adsorption rate	LH
r_d_ = *k*_d_θ_g_	Desorption rate	LH
r_g_ = *k*_g_C_g_θ_NiOOH_	Glucose reaction rate with surface	ER
r_int_ = *k*_int_θ_NiOOH_θ_g_	Intermediate formation	LH
r_p_ = *k*_p_θ_NiOOH_θ_int_	Product formation rate	ER and LH

Using the rate expressions in Table 1, we can write the surface coverage balance equations as

9 $$\frac{d\theta_{g}}{\mathrm{dt}}=r_{a}-r_{\mathrm{int}}-r_{d}$$

10 $$\frac{d\theta_{\mathrm{int}}}{\mathrm{dt}}=r_{g}+r_{\mathrm{int}}-r_{p}$$

11 $$\frac{d\theta_{\mathrm{NiOOH}}}{\mathrm{dt}}=r_{e}-\left(r_{g}+r_{\mathrm{int}}+r_{p}\right)$$

With the surface kinetics defined, we next couple the kinetics to mass and charge transport within the porous GLAD electrode to capture transport-limited behavior.

### 2.2 Mass and Charge Transport

In our electrochemical model, the nanocolumnar GLAD electrode is treated as a homogenized porous medium. The geometric features of the GLAD morphology are incorporated through specific surface area (SSA) and transport parameters including porosity (ϵ), and directional tortuosities (τ_i_). This formulation allows the governing equations to be solved on a 3D slab domain while still capturing the anisotropic transport behavior of the GLAD structure. Accordingly, the mass-transport governing equation can be written as

12 $$\frac{\partial C_{g}}{\partial t}=\underset{i}{\sum }\left(\frac{\epsilon }{\tau_{i}}D_{g}^{b}\right)\frac{\partial^{2}C_{g}}{\partial x_{i}^{2}}-\Gamma \cdot \mathrm{SSA}\cdot \left[r_{a}+r_{g}-r_{d}\right]$$

where D_g_^b^ is the bulk diffusion coefficient of glucose, and ϵ/τ_i_ accounts for porosity and directional tortuosity, yielding an effective diffusivity in direction i.^48^ Γ is the density of active sites per unit electrode area, and the term Γ·SSA converts surface reaction rates into a volumetric source/sink term within the porous electrode. Consistent with the dual-path mechanism, glucose is depleted by adsorption and direct reaction (r_a_ and r_g_) and returned by desorption (r_d_).

Charge transport in the electrolyte phase within the porous electrode is modeled by charge conservation, using an anisotropic effective conductivity and a source term that represents Faradaic current distributed over the internal surface area:

13 $$\underset{i}{\sum }\left(\frac{\epsilon }{\tau_{i}}\kappa^{b}\right)\frac{\partial^{2}\psi }{\partial x_{i}^{2}}=n_{e}\cdot \Gamma \cdot \mathrm{SSA}\cdot F\left(r_{f}|r_{b}\right)$$

where ψ is the electrolyte potential and κ^b^ is the bulk electrolyte conductivity. The factor ϵ/τ_i_ accounts for the morphology-induced reduction and anisotropy of ionic conduction, giving an effective conductivity along direction i. The right-hand side of the equation represents volumetric Faradaic charge transfer distributed over the internal surface area, where n_e_ is the number of transferred electrons, and (r_f_ – r_b_) is the net forward–backward Ni^2+^/Ni^3+^ redox rate.

eqs (9)–(12) are solved numerically using the finite element method (FEM) implemented in FEniCS (version 2019.1.0)^49–51^ on a 3D slab of size L_*x*_ = L_*y*_ = 1 μm and case-dependent thickness L_*z*_, discretized on a uniform 10 × 10 × 10 grid (with tetrahedral elements). Dirichlet boundary conditions are imposed on the top surface (C_g_ = C_g_^b^ and ψ = 0) while zero-flux (Neumann) conditions are applied on all remaining boundaries. As initial conditions, for both cyclic voltammetry and chronoamperometry, we start with no glucose or surface species present and a zero initial potential. The only difference is that cyclic voltammetry starts from a nonactivated surface (θ_NiOOH_ = 0), while chronoamperometry starts from a preactivated surface (θ_NiOOH_ = 1).

We also calculated the Faradaic current obtained by integrating the local electron-transfer rate throughout the porous electrode volume (Ω) as

14 $$I=\int_{\Omega }n_{e}\cdot F\cdot \Gamma \cdot \mathrm{SSA}{\cdot r}_{e}\cdot \mathrm{dV}$$

To apply the electrochemical model to specific GLAD morphologies, we require morphology-dependent inputs such as specific surface area and transport-related parameters. In the next section, we briefly describe the kMC simulations used to generate GLAD structures for use in the model.

### 3.3 Computational Details of kMC Simulation

The kMC simulation methodology and postprocessing are presented in full detail in our previous work.^17^ Here we briefly describe the method and include only salient details. kMC simulations of GLAD NiO growth were carried out using Stochastic Parallel PARticle Kinetic Simulator (SPPARKS),^52^ which accounts for both ballistic deposition and surface diffusion on an FCC lattice with a lattice parameter of d_0_, domain size of N_*x*_ × N_*y*_ × N_*z*_ and periodic boundary conditions in the x and y directions. A particle is released from a random point (x_0_, y_0_, z_0_) at the top of the domain and travels along a trajectory defined by the deposition angle (α) and the orientation of the substrate (ϕ). Ballistic capture is implemented by defining a cylinder of radius r_0_ around the incoming trajectory, and the first available site within this capturing distance becomes the deposition site.^53,54^ The deposition trajectory is defined as

15 $$\frac{x-x_{0}}{\tan\alpha \cos\phi }=\frac{y-y_{0}}{\tan\alpha \sin\phi }=z_{0}-z$$

In this kMC model, a site’s energy is considered as a linear function of the site’s coordination number. Thus, the Hamiltonian of the site i is described by

16 $$H_{i}=\underset{j}{\sum }\delta_{\mathrm{ij}}$$

where j represents the set of sites adjacent to i, and δ_ij_ are occupancy states. Here, when a neighboring site j is occupied, δ_ij_ is 1, and when the neighboring site is unoccupied δ_ij_ is 0.

In the GLAD growth simulation, we used a low surface diffusion approximation. In the experimental GLAD process, shadowing effects dominate over surface diffusion, leading to the formation of porous columnar films rather than dense compact coatings. High deposition rates also tend to be used in GLAD growth, implying that adatoms are not able to freely diffuse until optimum surface sites are found, but instead encounter one another, restricting migration. These conditions justify the low surface diffusion approximation as a physically reasonable choice for modeling GLAD growth. Additionally, adatom re-emission (via sputtering or evaporation) is not included in the present model, as under electron-beam evaporation and in the absence of the ion-bombardment-driven deposition, they are negligible and do not dominate the morphological evolution.^17,32^

#### 2.3.1 Post-Processing and Surrogate Model

The resulting simulated 3D GLAD structures are postprocessed to extract the surface area and transport-related parameters required by the electrochemical model. Each GLAD film is parametrized by SSA, ϵ, and τ_i_, which are then used to define anisotropic effective diffusivities and ionic conductivities. To extract these geometric features, we use the open-source Porous Microstructure Analysis (PuMA) framework,^55,56^ following the workflow described in our previous work.^17^ Final kMC configurations are voxelized into 3D image stacks. Porosity is computed from the void volume fraction,^57^ surface area is obtained via an iso-surface–based marching cubes algorithm,^58^ and the specific surface area is computed as the ratio of the structure’s total surface area to its total volume. To account for microstructure-induced transport limitations, we computed the tortuosity of the porous network using a random-walk,^54–56,58,59^

17 $$\tau_{i}=\epsilon \frac{D^{b}}{D_{i}^{e}}$$

Because running kMC simulations and characterization at every combination of deposition angle and substrate rotation rate is computationally expensive, we constructed a surrogate model that predicts morphological features. Using a data set generated by kMC, we treated (α, ϕ) as inputs and independently learned regression models for SSA, ϵ and τ_i_. Gaussian process regression (GPR) with standardized inputs was used to obtain smooth interpolants across the design space and to enable fast evaluation on dense (α, ϕ) grids. Model accuracy was quantified by comparing surrogate predictions to the reference data and standard regression metrics. The trained surrogate was then evaluated over the full parameter range to generate continuous heat maps of SSA, ϵ and τ_i_.

## 3 Experimental Method

### 3.1 Fabrication of GLAD Electrodes

GLAD NiO electrodes were prepared on masked Ni foils using a previously reported procedure.^29,30^ Briefly, Ni foil substrates (0.125 mm thick, ≥ 99.9% purity, Sigma-Aldrich Canada) were first cleaned (by bath sonication in acetone for 10 min followed by bath sonication in 18.2 MΩ·cm ultrapure water for 5 min) and dried under a nitrogen stream. The substrates were then masked with Kapton tape, exposing only a 0.28 cm^2^ area at one end of the substrate, and finally secured to an aluminum holder using Kapton tape. Depositions were carried out in a GLAD-enabled electron beam evaporation system (Kurt J. Lesker, USA) with computer-controlled motors that set and continuously adjust the substrate tilt and rotation. NiO pellets (2–3 mm, 99.97%, Materion Advanced Materials, USA) were loaded into graphite crucible liners, and the chamber was pumped down to a base pressure below 5 × 10^–5^ Pa. During NiO growth, the electron beam was operated at 7.5 kV and ∼ 80 mA, yielding a deposition rate of ∼ 0.3 nm/s at a working pressure near 3 × 10^–3^ Pa. To study the effect of deposition angle, Ni foils coated at incidence angles of 68°, 74°, and 80° relative to the substrate normal (previously reported in^17,19^ ) are reassessed here. To study the effect of substrate rotation, we performed additional depositions at 74° with three conditions: fixed substrate without rotation, slow rotation (2 turns/μm), and fast rotation (100 turns/μm). For scanning electron microscopy (SEM, Hitachi S-4800, Japan), pieces of Si wafers were also included in each GLAD process, and for ease of preparation and handling, these samples were used for SEM characterizations.

### 3.2 Electrochemical Measurement

Electrochemical testing of the GLAD-fabricated working electrodes was performed using a potentiostat (PalmSens, The Netherlands) in a three-electrode configuration, with Ag/AgCl as the reference electrode, a Pt coil as the counter electrode, and 0.25 M NaOH as electrolyte. The electrolyte was purged with N_2_ gas for 30 min prior to testing and was continuously blanketed with a stream of N_2_ throughout the measurement process. To activate the electrode surface, 125 cyclic voltammetry scans were recorded between 0 and 0.7 V using a scan rate of 20 mV/s. After activation, the glucose response of the GLAD NiO electrodes were evaluated by chronoamperometry at +0.55 V. First, a stabilization period of 200 s was performed, then glucose was added stepwise at 100 s intervals while gently stirring the solution at 350 rpm to achieve concentrations from 1 to 9 mM; this range was chosen to cover the clinically relevant glucose concentrations (3–8 mM).^16^ Sensitivity was then determined from the resulting chronoamperometric calibration data.

## 4 Results

### 4.1 kMC Simulation Results and Data Set

In our previous work, we validated our kMC simulation framework for electrodes composed of vertical posts by calculating porosity and surface area and benchmarking these results against both experimental and computational data available in the literature.^17,60,61^ We further demonstrated that morphological features such as porosity and tortuosity are independent of structure thickness and are governed solely by deposition angle and substrate rotation rate.^17,61^ In the present study, we extend this previous analysis of slanted structures (i.e., structures deposited by GLAD onto a fixed substrate) and vertical posts (i.e., structures deposited with high-speed substrate rotation) by examining a broader range of substrate rotation rates from 0 to 100 turns/μm, sampled at 18 discrete values. We also survey a range of deposition angles from 50° to 85° in 2.5° increments. The kMC simulation domain size is N_*x*_ = 75, N_*y*_ = 75, and N_*z*_ = 225 FCC lattice units with a lattice parameter of d_0_ = 0.417 nm. In total, 270 distinct structures were simulated, and for illustrative purposes, Figure 3 shows a subset of simulated structures formed at a deposition angle of 75° across various substrate rotation rates. As can be seen, intermediate rotation rates result in the formation of different helical geometries.

**Figure 3:**
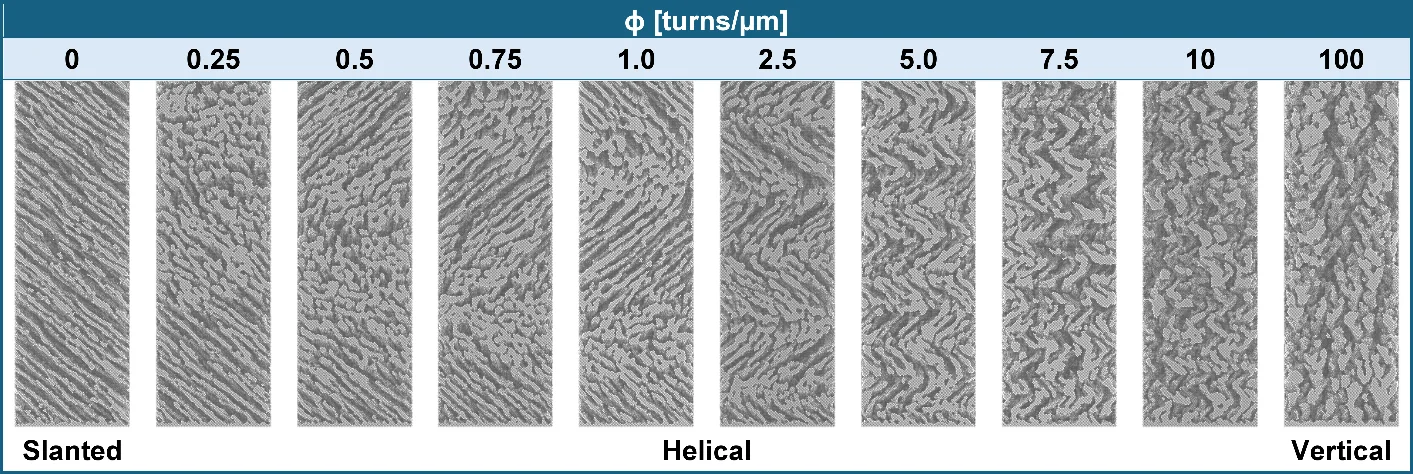
Sample of simulated structures formed at a deposition angle of 75° and different substrate rotation rates from 0 to 100 turns/μm.

#### 4.1.1 Geometric Features

After generating the kMC structure data set and extracting the geometric features, we used the surrogate model to predict porosity, specific surface area, and tortuosity across the full range of GLAD-fabricated morphologies. Figure 4 presents heat maps of specific surface area and porosity as functions of deposition angle and substrate rotation rate. At a fixed rotation rate, porosity (Figure 4.a) increases with deposition angle, because low angles produce limited shadowing and tightly packed GLAD columns, whereas higher angles increase shadowing, increase column spacing, and therefore increase porosity. In contrast, specific surface area (Figure 4.b) peaks around 60–65°, where the column density is highest, and the structure remains relatively compact, giving a large surface area. For angles above 65°, the reduced column density leads to lower total surface area. Substrate rotation rate also affects both metrics. At a fixed deposition angle, slow or zero rotation (slanted post regime) yields higher SSA than moderate or high rotation because the morphology contains a larger number of thinner columns. As the rotation rate increases, the number of columns decreases while individual columns become thicker, which reduces the total internal surface area (see Figure 3). The same trend is reflected in porosity, which generally decreases at moderate and high rotation rates relative to structures formed with slower rotation. It is worth noting that the kMC model was previously validated against experimental data in our prior work,^17^ combining qualitative comparison between the simulated structures and SEM images with quantitative validation of porosity and surface area against data reported in the literature.

**Figure 4:**
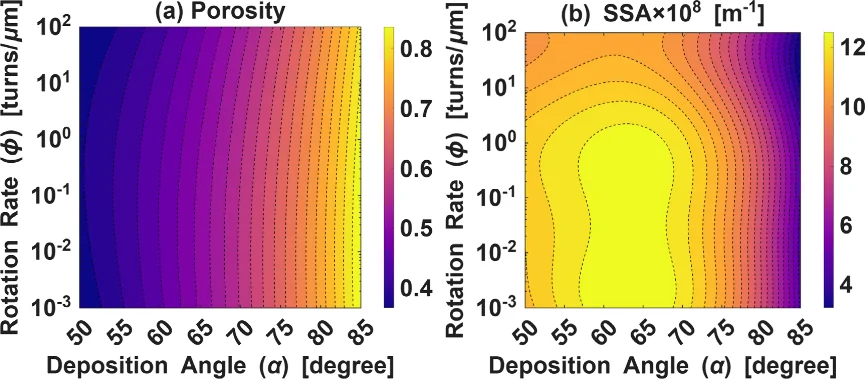
Heat maps of (a) porosity and (b) specific surface area for GLAD structures as functions of deposition angle and substrate rotation rate, obtained from the kMC-surrogate model.

It should be noted that the low surface diffusion approximation has a greater influence at lower deposition angles, where shadowing is weak and structures are more compact, possibly leading to a slight overestimation of porosity. As the deposition angle increases, self-shadowing dominates, and the effect of surface diffusion diminishes. A detailed validation of the present kMC scheme can be found in our prior work.^17^

Tortuosity is a key parameter controlling mass and charge transport in porous electrodes. To quantify transport in GLAD structures over a range of deposition angles and substrate rotation rates, we computed the directional tortuosities τ_*x*_, τ_*y*_, and τ_*z*_. Here, as shown in Figure 1, the *z*-direction is along the substrate normal, and the x-y directions lie in the plane of the substrate, with the x direction defined by the initial azimuthal direction of flux incidence during deposition. A slanted-post structure formed without substrate rotation is therefore inclined in the *x*–*z* plane. Tortuosity quantifies the winding of transport pathways within a porous medium, with lower tortuosity implying more direct pathways and less hindered transport. As a result, tortuosity typically decreases as porosity increases. Figure 5 shows heat maps of directional tortuosities of simulated GLAD structures as functions of deposition angle and substrate rotation rate. As can be seen, at a fixed rotation rate, τ_*x*_, τ_*y*_, and τ_*z*_ all decrease with increasing deposition angle. This behavior is consistent with stronger shadowing at higher angles, which increases column spacing and porosity and therefore facilitates molecular transport within the GLAD film. Regarding the effect of substrate rotation rate, high-speed rotation produces vertical posts (see Figure 3) and largely removes in-plane anisotropy, so τ_*x*_ and τ_*y*_ become nearly identical. In contrast, fixed and slow rotation can yield slanted or helical morphologies, leading to small differences between τ_*x*_ and τ_*y*_. Moreover, at a given deposition angle, τ_*x*_ and τ_*y*_ are slightly higher for vertical posts, consistent with their lower porosity compared to slanted and helical shapes. In contrast, τ_*z*_ is higher for fixed and slow rotation cases, where a larger number of thinner columns and smaller spacing create tighter pathways compared to vertical posts. Additionally, slanted and helical columns introduce more obstacles to transport in the z direction compared with vertical posts.

**Figure 5:**
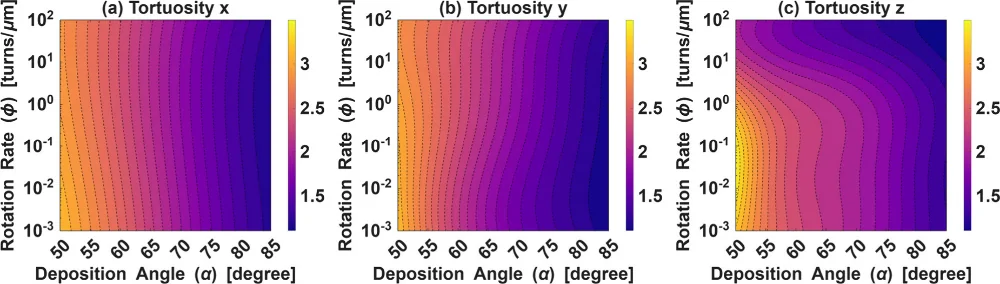
Heat maps of calculated directional tortuosities (a) τ_*x*_, (b) τ_*y*_, and (c) τ_*z*_ of GLAD structures as functions of deposition angle and substrate rotation rates, obtained from the kMC-surrogate model.

Figure 6 shows the ratios D_x_^e^/D^b^, D_y_^e^/D^b^, and D_z_^e^/D^b^, computed from the porosity and directional tortuosities (D_i_^e^/D^b^ = ϵ/τ_i_), as functions of deposition angle and substrate rotation rate for GLAD structures. Under the most limiting transport conditions (e.g., α = 60°), diffusivity can be reduced by 70–80%, while under the most transport-favorable conditions (e.g., α = 85°), the morphology reduces diffusivity by roughly 25–30%. Moreover, regarding the effect of substrate rotation, at a fixed deposition angle, low rotation rates generally lead to greater diffusivities due to the lower τ_*x*_ and τ_*y*_ compared with higher rotation rates, as shown in Figure 5. In contrast, D_z_^e^ increases with rotation rate because τ_*z*_ decreases as the structure transitions toward a vertical shape. These results highlight the importance of incorporating morphology-dependent transport properties when analyzing and designing GLAD electrodes.

**Figure 6:**
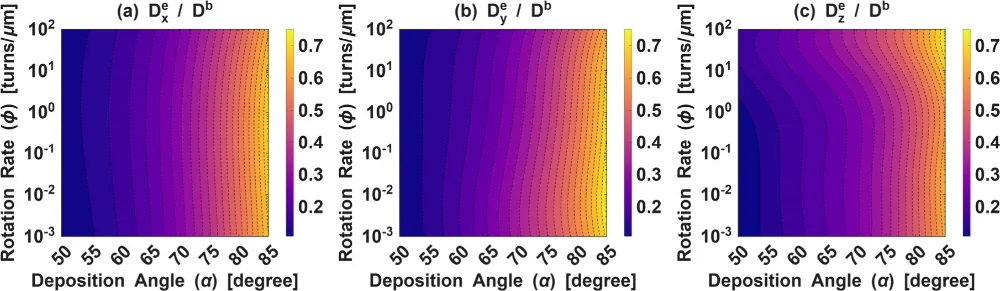
Heat maps of calculated directional diffusivities in GLAD structures as functions of deposition angle and substrate rotation rates, obtained from the kMC-surrogate model.

### 4.2 Electrochemical Model Calibration, Validation, and Prediction

Following the calculation of geometric features and their integration into the developed electrochemical model, the next step is to use experimental data to calibrate the model and determine model parameters and rate constants. For this purpose, we used both experimental and simulated cyclic voltammetry (CV) data for a vertical posts GLAD structure deposited at a 74° deposition angle with a thickness of 950 nm presented in.^16^ First, to calibrate the density of active sites (Γ) and the parameters in eq (8) (*k*_f_, *k*_b_, β, and E_eq_), we used the CV of the Ni^2+^/Ni^3+^ redox pair at a scan rate of 10 mV/s in the absence of glucose. Next, to calibrate the rate constants *k*_a_, *k*_d_, *k*_g_, k_*int*_, and *k*_p_ appearing in the rate expressions in Table 1, we used CV results at a scan rate of 10 mV/s in the presence of 3 mM glucose in the electrolyte. The fitting was performed by minimizing the root-mean-square error between simulated and experimental CVs using a two-stage approach: first, a global optimization (differential evolution) followed by local refinement. Figure 7 and Table 2 show the corresponding fit results and parameters. Because the model does not account for water splitting, we performed the fitting only up to 0.55 V as indicated by the dashed lines in Figure 7.

**Figure 7:**
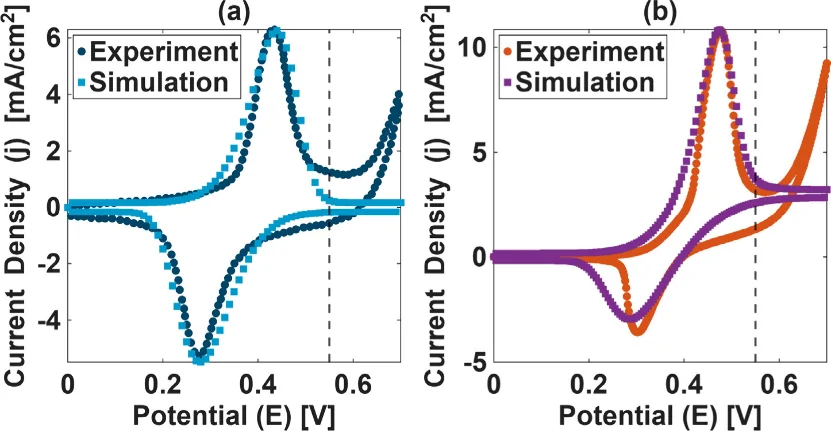
Fitted cyclic voltammograms at a scan rate of 10 mV/s for vertical GLAD NiO (deposition angle of 74°, thickness 950 nm) in 0.25 M NaOH: (a) 0 mM glucose and (b) 3 mM glucose using experimental data from a previous study.^16^ The dashed line indicates the maximum potential used for fitting.

**Table 2:** Kinetic Rate Constants and Model Parameters Used in the Simulations^a^

**Rate Constants**	**Value**	**Other Parameters**	**Value**
*k*_f_ [s^–1^] *	0.04	*k*_p_ [s^–1^] *	1.0
*k*_b_ [s^–1^] *	0.05	Γ [mol·m^–2^] *	9.3 × 10^–6^
*k*_a_ [mM^–1^·s^–1^] *	2.5 × 10^–3^	β *	0.54
*k*_d_ [s^–1^] *	2.5 × 10^–3^	E_eq_ [V] *	0.35
*k*_g_ [mM^–1^·s^–1^] *	8.069 × 10^–3^	D_g_^b^ [m^2^·s^–1^][Bibr ref62]	6 × 10^–10^
*k*_int_ [s^–1^] *	1.0	κ^b^ [S·m^–1^][Bibr ref63]	5

a Parameters marked with (*) were fitted as shown in Figure 7, and the two remaining parameters were taken from the references indicated.

Using the calibrated parameters, we evaluated the chronoamperometric responses of both simulated and experimental vertical-post GLAD NiO electrodes fabricated at 74° deposition angles and 1550 nm thicknesses (shown in Figure 2) to successive 1 mM glucose additions every 100 s in 0.25 M NaOH at an applied potential of 0.55 V (under conditions similar to those described in Section 3.2 and^16^). As shown in Figure 8, the simulation closely reproduces the experimental response, indicating that the model captures the reaction-transport behavior.

**Figure 8:**
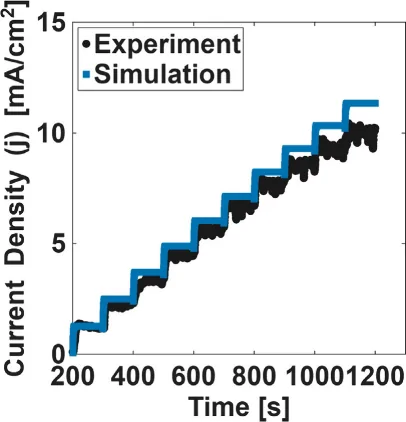
Chronoamperometric response of GLAD NiO (deposition angle of 74°, thickness 1550 nm) to successive increase of 1 mM glucose concentration every 100 s in 0.25 M NaOH at an applied potential of 0.55 V, comparing simulated and experimental currents.

The calibrated parameters were subsequently used to predict the sensitivity across all analyzed GLAD NiO morphologies, simulated over deposition angles of 60–85° and substrate rotation rates of 0–100 turns/μm at a fixed thickness of 1500 nm, as shown in Figure 9a. Model predictions were then compared with experimentally measured sensitivities for the representative structures in Figure 2, with the experimental values summarized in Figure 9b. It can be seen that the model captures both the trend and the magnitude of the measured sensitivities. For the vertical structures (substrate rotation speed of 100 turns/μm), both the model and experiments show sensitivity peaks with deposition angle. The model peaks at 72.5°, and the experiments peak at 74°, indicating close agreement. Although the maximum specific surface area occurs at lower deposition angles (60–65°, as shown in Figure 4), the optimal sensitivity shifts to higher angles because mass transport limitations become notable at low deposition angles in dense, low-porosity structures. In that region, the high directional tortuosities reduce the effective diffusivities substantially (with roughly 70–80% of the bulk diffusivity suppressed), pushing the system into a transport limited regime and lowering sensitivity despite the higher surface area. Increasing the deposition angle to 72.5–74° improves porosity and reduces tortuosity, yielding an optimal balance between accessible surface area and mass transport limitation. This finding aligns with experimental observations indicating that, although the 68° vertical post has a higher surface area, it does not yield the highest sensitivity. In contrast, the 74° vertical structure demonstrates higher sensitivity. At deposition angles greater than 72.5°, mass transport limitations decrease; however, the significant reduction in surface area leads to fewer active sites, resulting in decreased sensitivity. For variations in substrate rotation rate, the model predicts that slanted posts (i.e., no rotation) exhibit the highest overall performance, followed by helical structures and then vertical posts. This prediction contrasts with our long-standing intuition and suggests an electrode design that improves upon all of our previous experimental studies of GLAD-based analytical sensors. Specifically, whereas a recent study employed a vertical GLAD NiO morphology deposited at 74°^16^ our model indicates that a slanted post morphology at the same deposition angle should deliver higher overall performance. This prediction is consistent with experimental observations shown in Figure 9.b, where a slanted post structure deposited at 74° improves electrode sensitivity by approximately 25% with respect to the previously optimum vertical posts structure (also formed at 74°). As predicted by the model, the experimentally measured helical structure represents an intermediate case, with glucose sensitivity ∼ 15% greater than the vertical-posts structure. This trend is primarily attributed to morphology-dependent surface area and column density. Configurations with a greater number of thinner columns (i.e., slanted posts) offer a larger total surface area compared to those with fewer, thicker columns (i.e., vertical posts). Therefore, although vertical posts may be slightly less limited by transport, their lower total surface area, relative to fixed substrate and slow rotation cases, results in reduced sensitivity.

**Figure 9:**
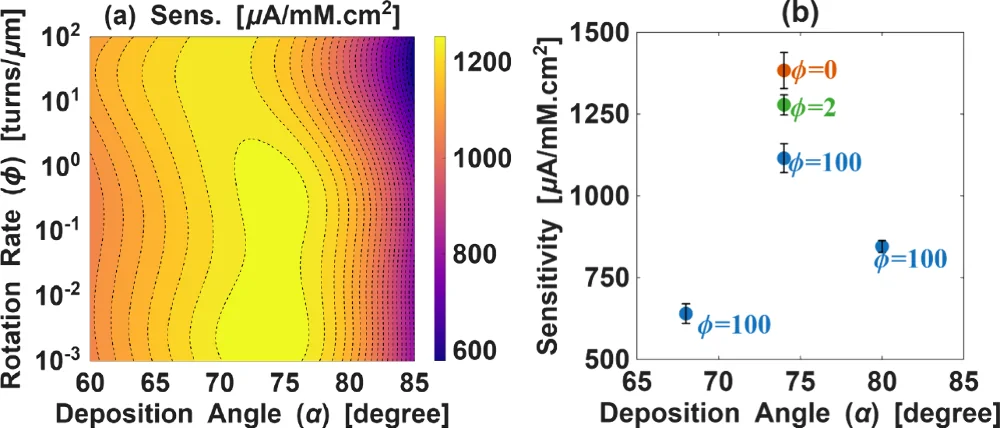
Experimental and computed glucose sensitivities of GLAD NiO electrodes as a function of deposition angle and substrate rotation rate at a fixed thickness of 1500 nm: (a) model-predicted sensitivity heat map over α = 60–85° and ϕ = 0–100 turns/μm, and (b) experimentally measured sensitivities for the representative structures shown in Figure 2. For each structure, two independent standard-addition series (each spanning 0–10 mM glucose) were combined into a single linear regression; the sensitivity is the fitted slope, and the error bars represent the standard error of that slope. The measurements show a peak at α = 74° and ϕ = 0 turns/μm, in good agreement with the model trends.

As mentioned previously, the low surface diffusion approximation tends to slightly overestimate the surface area, possibly leading to slightly overestimated sensitivity values, particularly at lower deposition angles (<60°); however, it does not affect the relative performance ranking of the structures or the optimal design.

## 5 Discussion

With the kMC framework developed in this work, we quantify key morphology dependent parameters (surface area, porosity, and tortuosity) for a wide range of GLAD structures including slanted posts, helical morphologies, and vertical nanocolumns, across a broad space of deposition angles and substrate rotation rates. By incorporating the geometric features into a coupled reaction-transport electrochemical model, we can predict the GLAD-fabricated electrode performance, at lower cost and time penalty than through experimental investigation. To the best of our knowledge, such an integrated performance and optimal design prediction workflow has not previously been reported for GLAD-fabricated electrodes. A key insight from this study is that, although GLAD electrode morphologies provide high surface area, their performance can be strongly constrained by mass transport limitations. The effective diffusivity within the porous medium is substantially reduced relative to the bulk value (20–80%), which can affect the benefit of the high surface area. Consistent with these findings, our modeling proposes a new GLAD NiO electrode design that maximizes glucose electro-oxidation by achieving an optimal trade-off between available surface area and mass transport limitations: slanted posts deposited at an incidence angle of 72.5° were predicted to achieve this optimum. The predicted design was then tested experimentally and found to improve GLAD-fabricated electrode performance by ∼ 25% relative to previously used designs (i.e., the sensitivity of the slanted post structure was measured to be 1.38 mA/mM·cm^2^ vs 1.12 mA/mM·cm^2^ for the vertical posts).

The main limitations of the present framework are that the kMC growth model employs an on-lattice, low diffusion limit appropriate for high deposition rates during thin film growth, neglecting surface diffusion and potential amorphous growth effects. Future studies could incorporate surface diffusion and an off-lattice deposition to the model to achieve more realistic growth processes. Also, this analysis considers only a single analyte system (glucose), whereas future work could extend the electrochemical formulation to multianalyte sensing relevant to diabetes monitoring or other diagnostic assays. Such a study would require incorporating multispecies transport as well as competitive adsorption/desorption and reaction kinetics to capture potential interference effects from other biomolecules present in real biofluids.

More broadly, the workflow can be readily extended to other analytes by substituting the appropriate reaction mechanism and kinetic parameters and by updating analyte transport properties. These changes are expected to shift the optimal GLAD morphology depending on whether sensor performance is primarily kinetics limited (favoring higher surface area obtained at lower deposition angles) or transport limited (favoring higher porosity, obtained at higher deposition angles). Additionally, the same coupled kMC–electrochemical modeling framework can be applied to other GLAD-fabricated electrode materials by updating material-specific growth inputs (crystal structure and lattice parameters) and electrocatalytic kinetics, enabling applications beyond glucose sensing, including broader electrochemical technologies such as energy conversion and hydrogen generation. For energy applications such as hydrogen generation, additional complexities arise, including differences in operating potentials, electrolyte conditions and gas bubble formation that are not captured in the current transport model. Despite the challenges, the core workflow of linking GLAD fabrication input parameters to electrochemical performance through kMC-generated morphology, surrogate modeling, and a homogenized porous electrode model, provides an efficient route for exploring these other GLAD applications. This approach potentially significantly reduces experimental trial-and-error by enabling efficient and rapid testing of fabrication and structural parameters.

## 6 Conclusion

In this work, we presented a multiscale modeling framework that directly links the fabrication parameters of GLAD-fabricated NiO electrodes including deposition angle and substrate rotation rate, to their electrochemical performance in nonenzymatic glucose sensing. By combining kMC-based GLAD growth simulations with surrogate modeling, we calculated geometric features such as surface area, porosity, and directional tortuosity to understand how deposition angle and substrate rotation rate influence electrode morphology. To investigate glucose electro-oxidation, we then incorporated the extracted geometric features into a coupled reaction–transport electrochemical model that integrates a dual-path kinetic mechanism along with charge and mass transport within the porous GLAD electrode. Our results indicate that the deposition angle of 72.5° maximizes glucose electro-oxidation by balancing surface area enhancement and mass transport limitations, even though it does not provide the highest surface area. Furthermore, we found that slanted posts outperform helical shapes, and helical shapes are superior to vertical posts at a given deposition angle for glucose electro-oxidation. This is a new observation, and we found that slanted-post electrodes fabricated based on this concept were approximately 25% more sensitive to glucose than the previously reported vertical posts morphologies. This is because configurations with a greater number of thinner columns (slanted posts) offer a larger total surface area compared to those with fewer, thicker columns (vertical posts). Although demonstrated here for GLAD NiO electrodes and glucose sensing, the proposed modeling framework provides a predictive approach for designing GLAD-fabricated materials beyond NiO, and across a broad range of applications such as energy conversion and hydrogen generation systems. This approach significantly reduces experimental trial-and-error by enabling efficient and rapid testing of fabrication and structural parameters.

## Supplementary Material



## Notes

FEM mesh convergence analysis; surrogate model validation and cross-validation results; calibration curves for slanted and helical GLAD NiO electrodes; dual-path kinetic model fitting details (PDF)
