# Supplementary material for: Model-Driven Discovery of Highly Sensitive Nanocolumnar NiO Electrochemical Glucose Sensors
Source: ACS Eng Au. 2026 Jul 16;6(4):567–77. doi: 10.1021/acsengineeringau.6c00026 (PMC13495675; doi:10.1021/acsengineeringau.6c00026)
Supplement: Supplementary file 1 [file eg6c00026_si_001.pdf]

Supporting Information for Model-Driven Discovery of Highly Sensitive Nanocolumnar NiO Electrochemical Glucose Sensors

*Authors: MohammadAli Maleki Bigdeli [a, *], Jennifer Bruce [b], Abebaw B. Jemere [b, c], Kenneth D. Harris [a, b], and Wylie Stroberg [a, e *]*

[a] Department of Mechanical Engineering, University of Alberta, Donadeo Innovation Centre for Engineering, 116 St NW, Edmonton, Alberta, T5K 1S6, Canada.

[b] Quantum and Nanotechnologies Research Centre, National Research Council Canada, 11421 Saskatchewan Dr NW, Edmonton, Alberta, T6G 2M9 Canada.

[c] Department of Chemical and Materials Engineering, University of Alberta, Donadeo Innovation Centre for Engineering, 116 St NW, Edmonton, Alberta, T5K 1S6, Canada.

[e] Department of Biomedical Engineering, University of Alberta, Donadeo Innovation Centre for Engineering, 116 St NW, Edmonton, Alberta, T5K 1S6, Canada.

[*] Corresponding authors: E-mail address: alibigdeli@ualberta.ca (MohammadAli Maleki Bigdeli), stroberg@ualberta.ca (Wylie Stroberg).

1 Finite Element Model Mesh Convergence

To determine the optimal mesh resolution for our finite element simulations of our reaction-diffusion model, a mesh convergence study was performed for the vertical GLAD structure at a deposition angle of 75°. We tested mesh resolutions from N = 10 to N = 30 elements per direction and compared the calculated sensitivity of the electrochemical sensor at each resolution. Then, errors relative to the finest mesh (N=30 elements per direction) were calculated. As shown in Figure S.1, they remain below 0.1% in all cases. Therefore, a uniform 10×10×10 grid (with tetrahedral elements) was used for all simulations to reduce computational cost while maintaining accuracy.

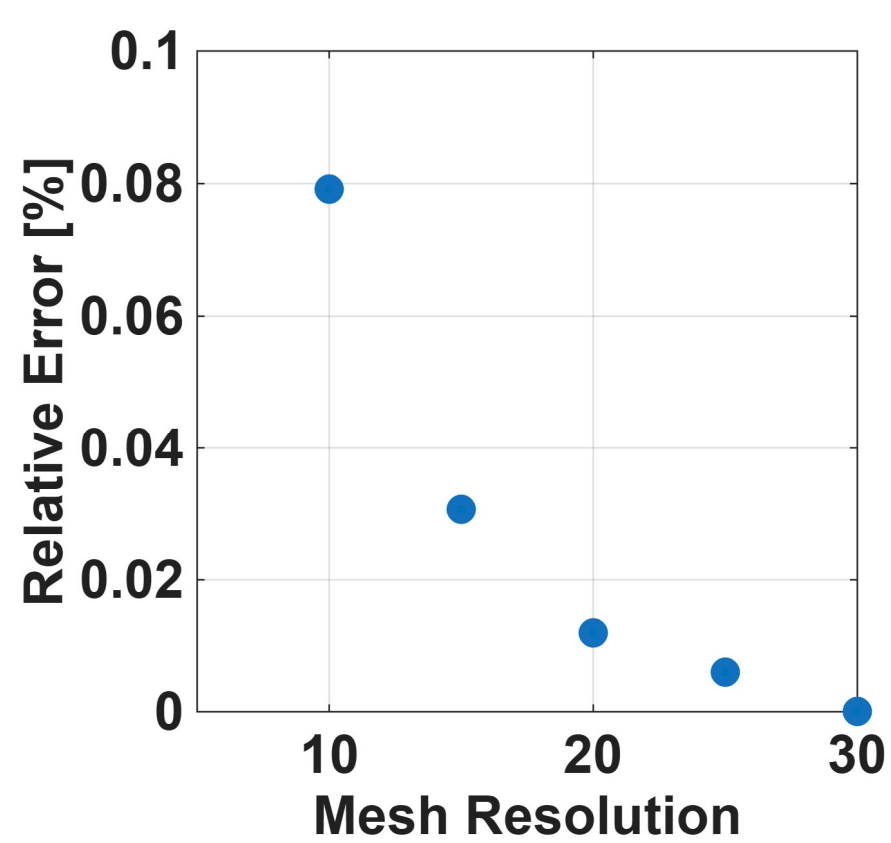

Figure S1 – Mesh convergence analysis for the FEM model, showing the relative error in sensitivity as a function of mesh resolution for a vertical GLAD structure deposited at a 75° angle.

2 Surrogate Model Validation

To further analyze the accuracy of the surrogate model, parity plots that compare Gaussian process regression (GPR) prediction with the kMC-generated datasets are presented in Figure S2-Figure S5. Each point corresponds to a specific combination of deposition angle (α) and substrate rotation rate (ϕ). The surrogate model was trained using α and ϕ as input features with separate GPR models for specific surface area (SSA), porosity (ϵ), directional tortuosities (τ_x, τ_y, τ_z),

directional diffusivities (D_x^e, D_y^e, D_z^e). Also, sensitivity values were first calculated using FEM simulations for all structures in our dataset and then similar to other parameters, we trained a GPR surrogate model to enable sensitivity prediction. As shown in Figures S2-S5, excellent agreement is observed between the predicted and true morphological features, directional tortuosities, and sensitivity, with $R^2 > 0.998$ in all cases.

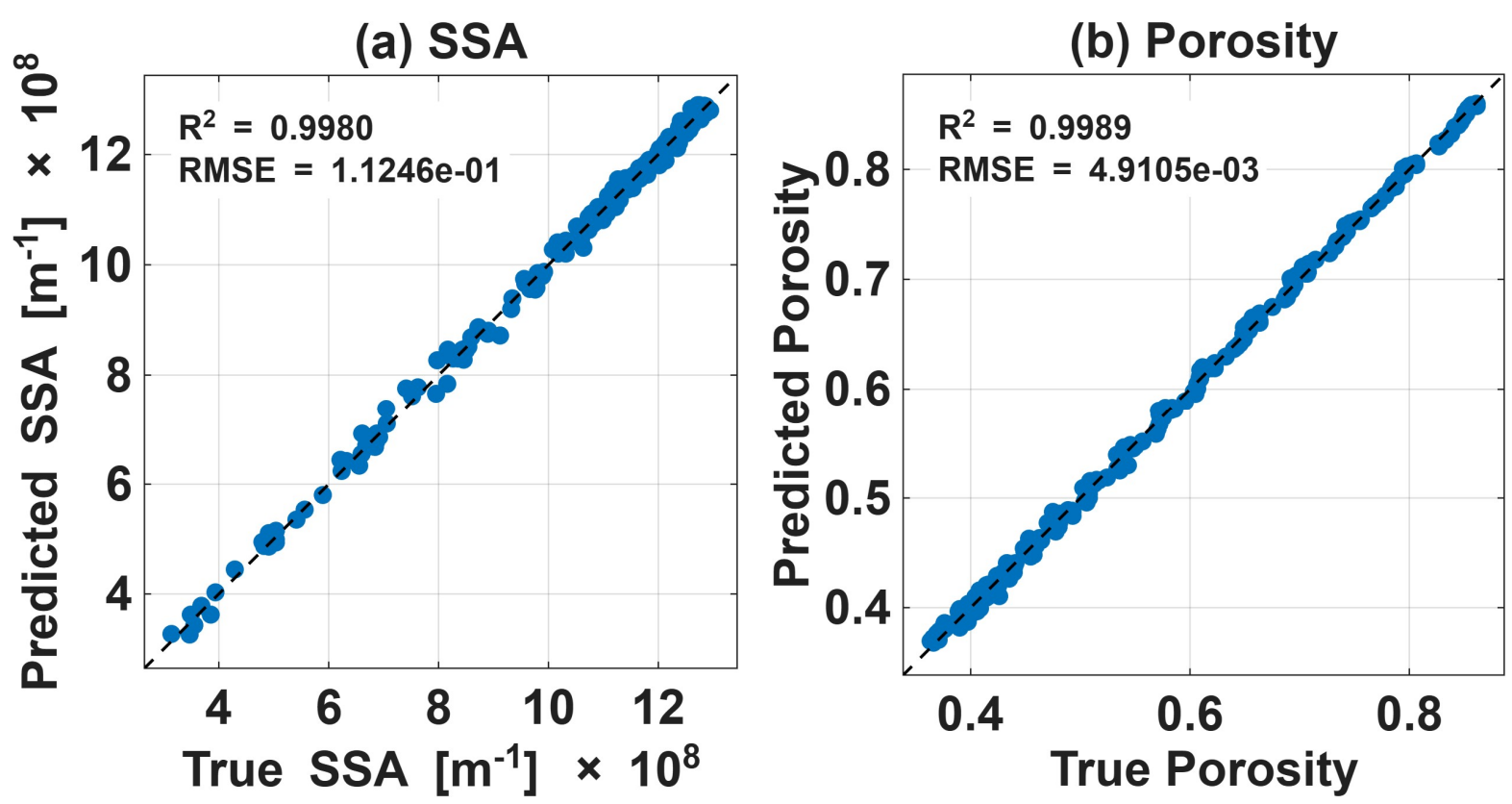

Figure S2 – Comparison of GPR predictions and kMC-generated data for (a) specific surface area (SSA) and (b) porosity.

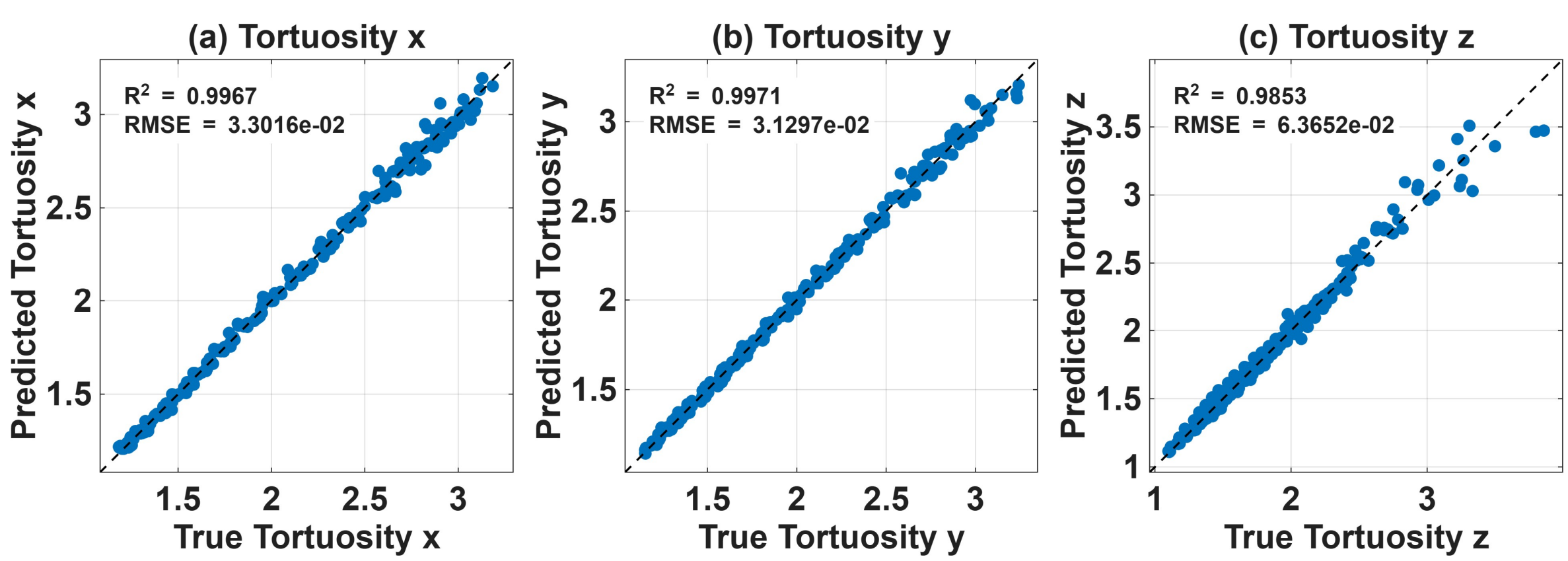

Figure S3 – Comparison of GPR predictions and kMC-generated data for directional tortuosities (τ_x, τ_y, τ_z).

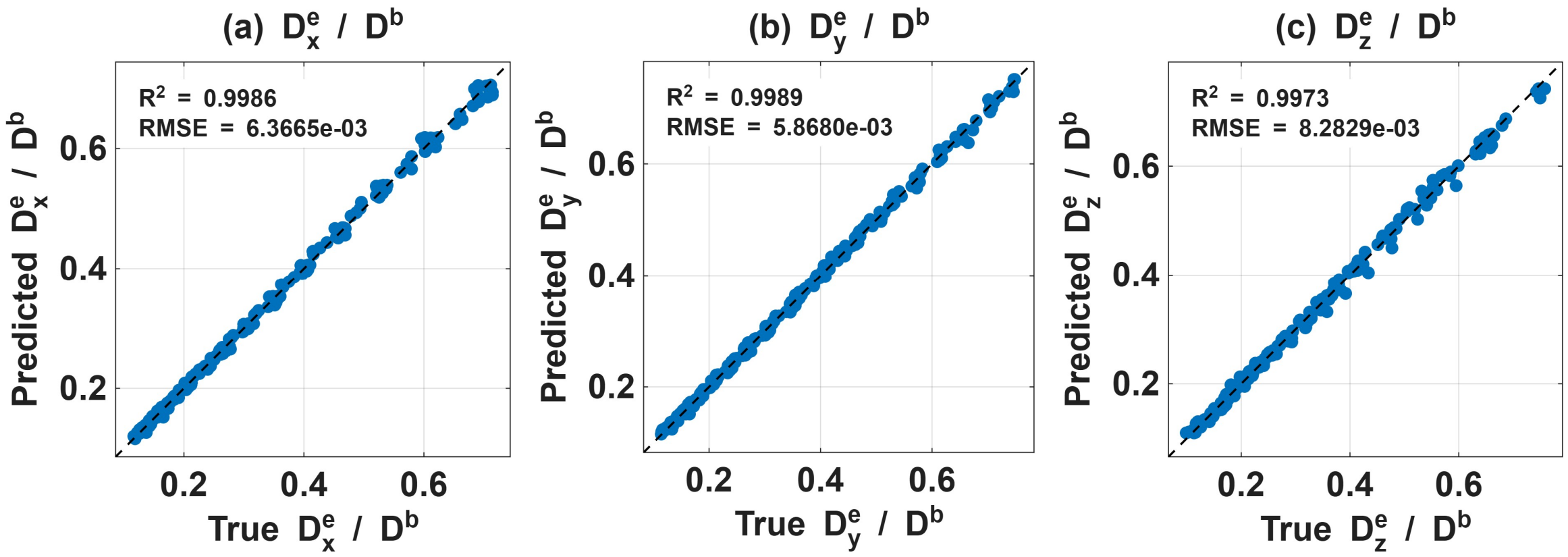

Figure S4 – Comparison of GPR predictions and kMC-generated data for directional diffusivities (D_x^e, D_y^e, D_z^e).

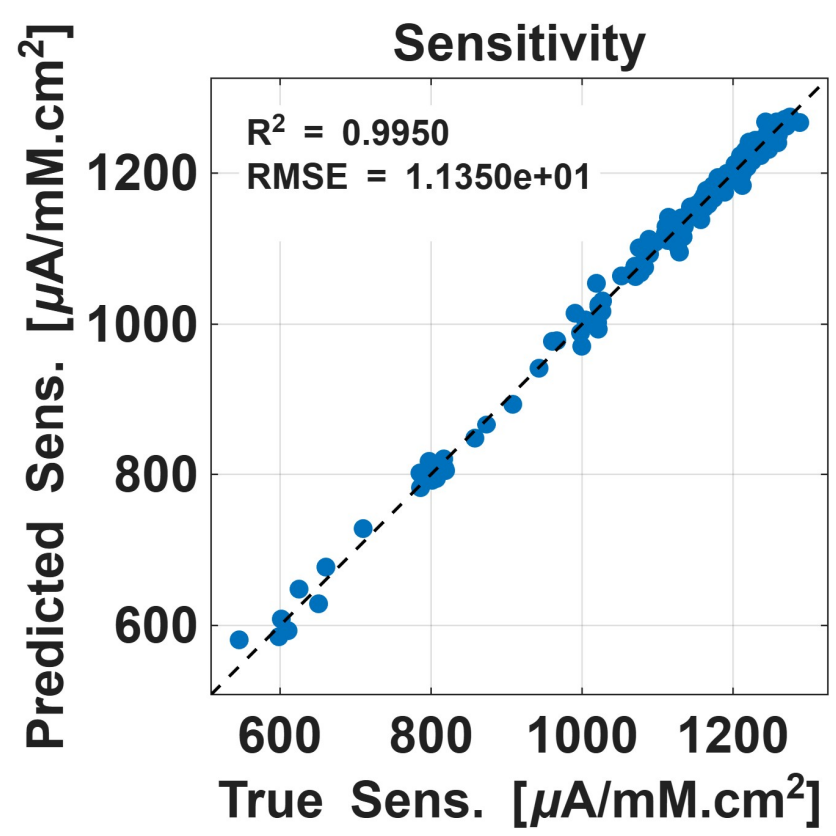

Figure S5 – Comparison of GPR predictions and FEM simulation results for electrochemical sensitivities.

To better evaluate the robustness of the surrogate models, we used a 10-fold cross-validation procedure on the full kMC dataset. We trained the GPR model on 90% of the data in each round and tested it on the remaining 10%. The predictions from each round are shown in Figures S6-S9. As can be seen, the cross-validation metrics closely match the training metrics, showing reliability and accuracy of the surrogate models.

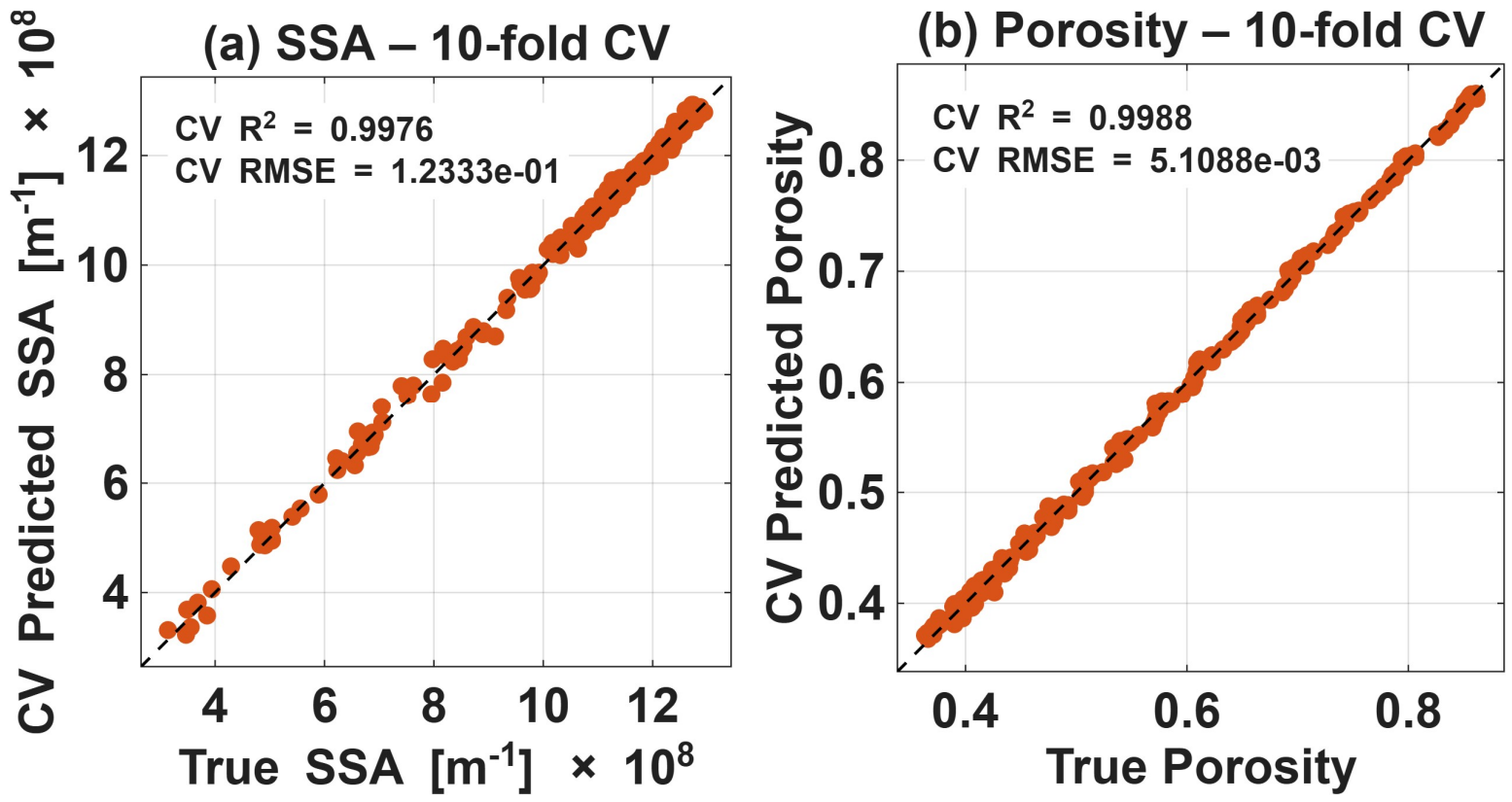

Figure S6 – Comparison of GPR predictions and kMC-generated data for (a) specific surface area (SSA) and (b) porosity.

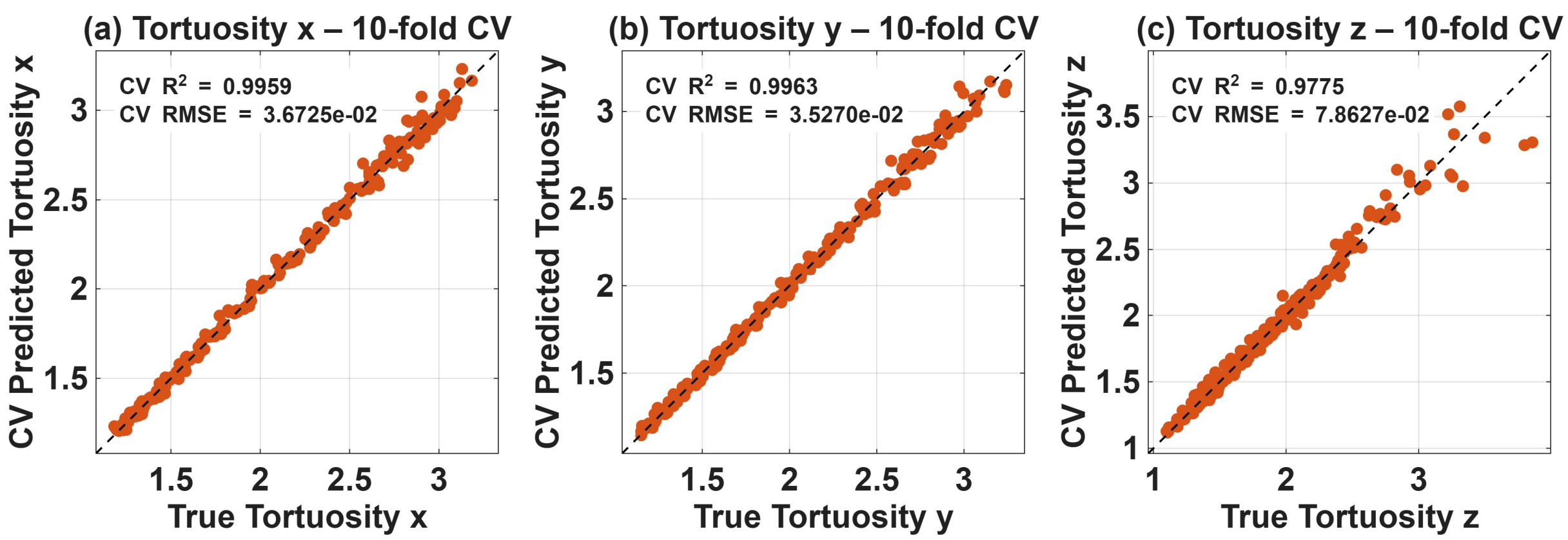

Figure S7 – Comparison of 10-fold cross-validation GPR predictions and kMC-generated data for directional tortuosities (τ_x, τ_y, τ_z), found in panels (a), (b), and (c), respectively.

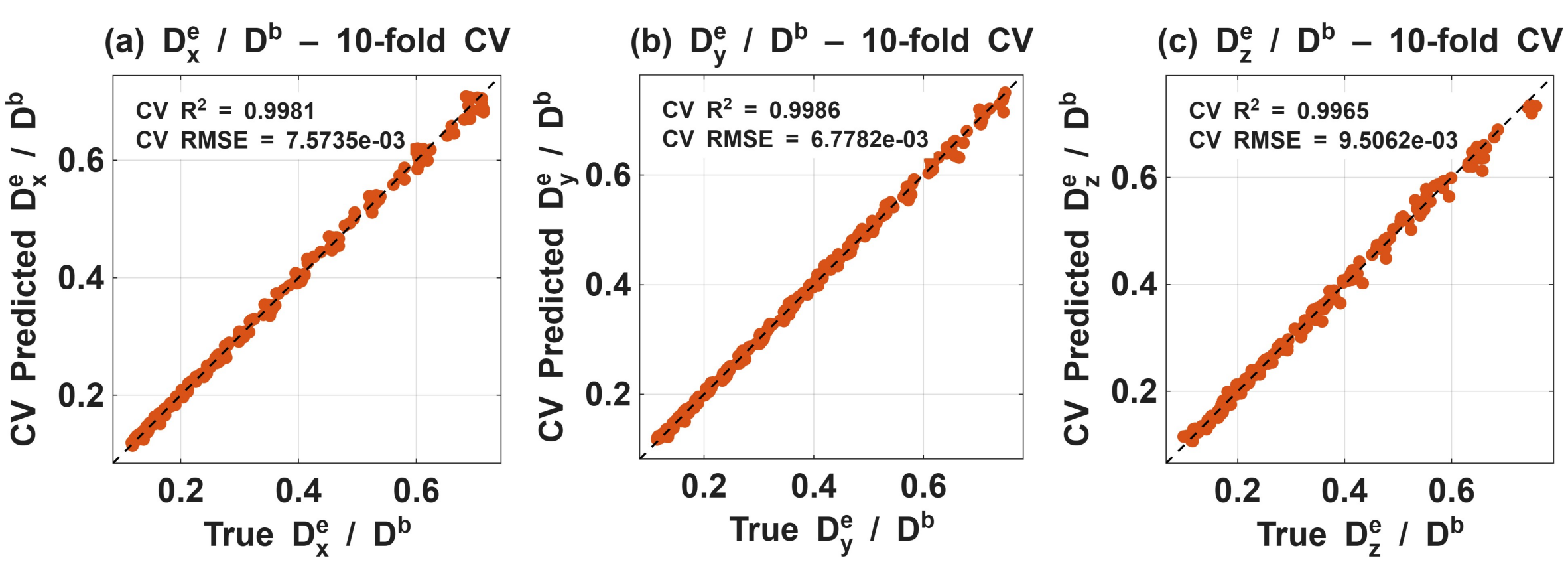

Figure S8 – Comparison of 10-fold cross-validation GPR predictions and kMC-generated data for directional diffusivities (D_x^e, D_y^e, D_z^e), found in panels (a), (b), and (c), respectively.

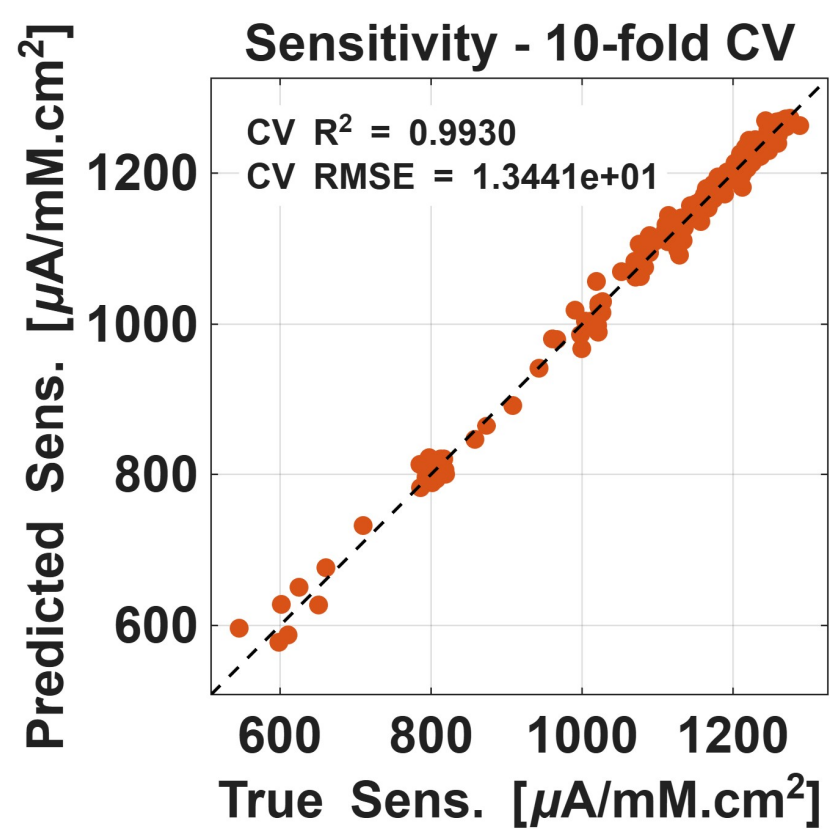

Figure S9 – Comparison of 10-fold cross-validation GPR predictions and FEM simulation results for electrochemical sensitivities.

3 Calibration Curves for Slanted and Helical GLAD Electrodes

In addition to the vertical-post electrode presented in the main manuscript, slanted-post (0 turns/μm substrate rotation) and helical (2 turns/μm substrate rotation) morphologies were experimentally characterized and compared with model predictions. The corresponding calibration curves are shown in Figure S10.

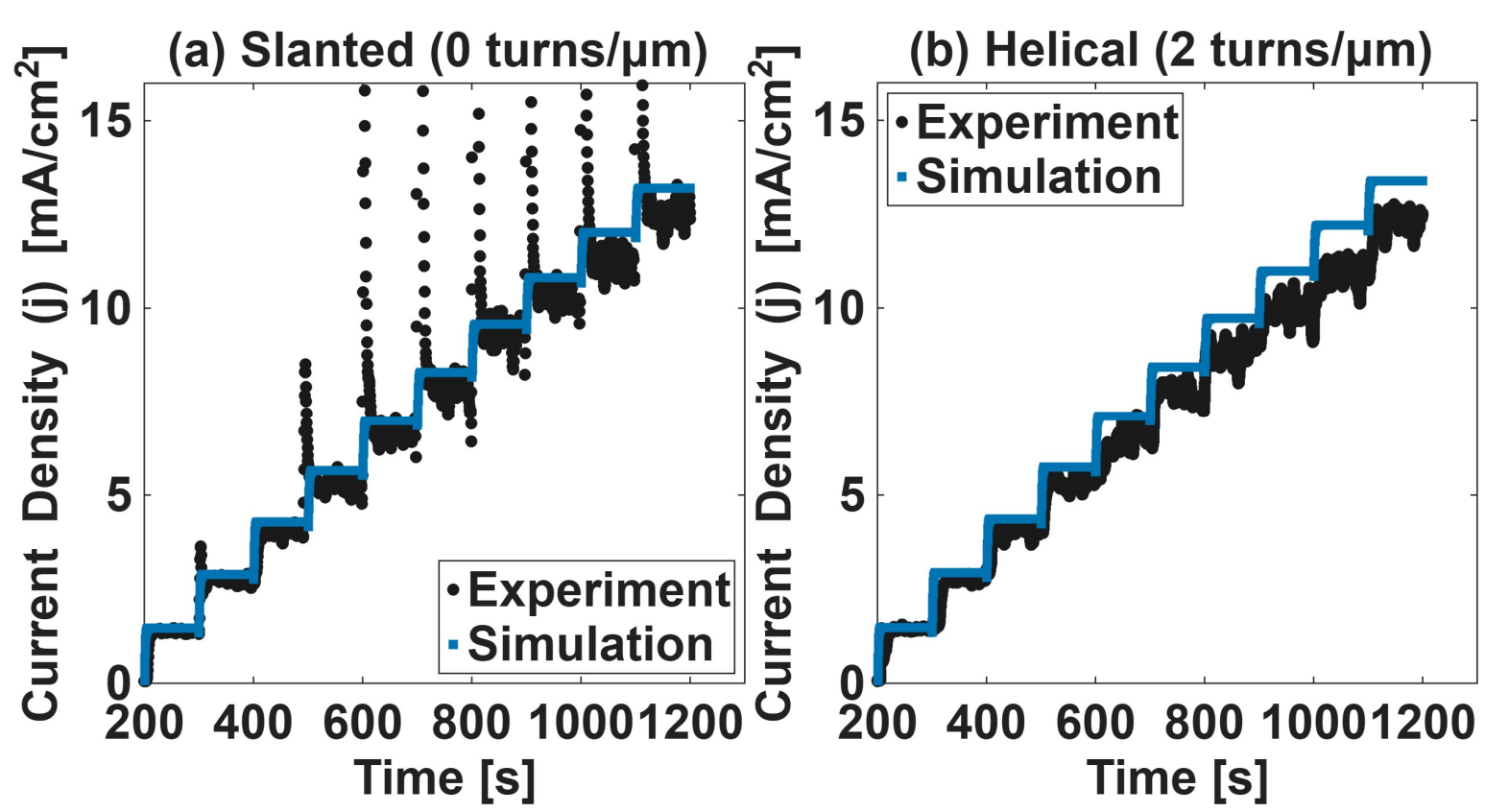

Figure S10 – Chronoamperometric responses of (a) slanted (0 turns/μm substrate rotation) and (b) helical (2 turns/μm substrate rotation) GLAD NiO electrodes, both deposited at a deposition angle of 74° and a film thickness of 1550 nm, to successive increase of 1 mM glucose concentration

every 100 s in 0.25 M NaOH at an applied potential of 0.55 V, comparing simulated and experimental currents.

4 Dual-path kinetic model fitting

Model parameters were determined using a two-stage optimization process that combined global and local methods to identify and refine parameter sets that agreed well with experimental data.[1] First, to calibrate the density of active sites (Γ), forward and backward rate constants for the Ni redox step (k_f, k_b), charge transfer coefficient (β) and the equilibrium potential of the Ni^{2+}/Ni^{3+} redox pair (E_{eq}), we used the CV of the Ni^{2+}/Ni^{3+} redox pair at a scan rate of 10 mV/s in the absence of glucose. Next, to calibrate the rate constants k_a, k_d, k_g, k_{int}, and k_p appearing in the rate expressions in the paper's Table 1, we used CV results at a scan rate of 10 mV/s in the presence of 3 mM glucose in the electrolyte. The fitting process was implemented in Python using a coupled FEM and optimization algorithm. An initial parameter space was explored using differential evolution using the SciPy implementation (with strategy = "best1bin", maxiter = 60, popsize = 16, mutation = (0.5, 1.0), recombination = 0.7, and seed = 1), followed by local refinement using Nelder–Mead and then L-BFGS-B algorithms, with the initial guess taken from the best result of the differential evolution step. The objective function was defined as the root mean square (RMSE) error between the simulated and experimental current densities, evaluated over the potential window $E \leq 0.55$ V. These results were then used to guide manual adjustments to further improve agreement with experimental trends. Based on prior studies[2,3] and model behavior, k_{int} and k_p were fixed 1 s^{-1} (considering the 3 mM glucose concentration) during the manual adjustment to make sure r_{int} and r_p are higher than r_a, r_g and r_d (by approximately one to two orders of magnitude) to

ensure that intermediate steps do not limit the kinetics and the surface is not poisoned.[3] Also, based on our observations, when k_g is relatively higher than k_a (at least one order of magnitude higher), the cathodic peak in the CV plot disappears, whereas a lower k_g leads to underestimation for the anodic current at higher potentials. Therefore, k_a and k_g were constrained to remain of the same order of magnitude by allowing both to vary within predefined bounds during optimization. Overall, k_g controls the magnitude of the anodic current, whereas the ratio of k_a and k_g governs the overall shape of the CV curve. More details on the model calibration can be found in this study.[2]

5 References

(1) Singer, N.; Pillai, R. G.; Johnson, A. I. D.; Harris, K. D.; Jemere, A. B. Nanostructured Nickel Oxide Electrodes for Non-Enzymatic Electrochemical Glucose Sensing. *Microchim Acta* **2020**, *187* (4), 196. https://doi.org/10.1007/s00604-020-4171-5.

(2) Medrano-Banda, A.; Guehl, J.; Kéranguéven, G.; Oshchepkov, A.; Savinova, E.; Bonnefont, A. Dual-Path Glucose Electrooxidation Reaction on Ni(OH)2/NiOOH Catalysts in Alkaline Media. *Electrochimica Acta* **2024**, *476*, 143692. https://doi.org/10.1016/j.electacta.2023.143692.

(3) Fleischmann, M.; Korinek, K.; Pletcher, D. The Oxidation of Organic Compounds at a Nickel Anode in Alkaline Solution. *Journal of Electroanalytical Chemistry and Interfacial Electrochemistry* **1971**, *31* (1), 39–49. https://doi.org/10.1016/S0022-0728(71)80040-2.
